# Systematic review of lung function assessment among youth and young adults e-cigarette users: Current tools and emerging methods

**DOI:** 10.1371/journal.pone.0342500

**Published:** 2026-02-06

**Authors:** Nirul Isamuddin Nasir, Mohd Hasni Ja’afar, Norfazilah Ahmad

**Affiliations:** Department of Public Health Medicine, Faculty of Medicine, Universiti Kebangsaan Malaysia, Cheras, Kuala Lumpur, Malaysia; Srebrnjak Children's Hospital, CROATIA

## Abstract

**Introduction:**

This review focuses on the need to identify the lung function assessment tools used for young EC users. The objectives are to examine the current and emerging methods used in assessing lung function among young EC users, besides identifying the alterations in lung function following EC exposure measured by those tools.

**Methodology:**

This systematic review was conducted according to the Preferred Reporting Items for Systematic Reviews and Meta-Analyses (PRISMA) 2020 flow checklist. Six databases (Web of Science, PubMed, Scopus, Taylor & Francis, SAGE, and ScienceDirect) were searched in April 2025 for original articles published between 2016 and 2025. Quality appraisal of the eligible articles was conducted using the Joanna Briggs Institute (JBI) Critical Appraisal Tools. Findings were synthesized using Narrative analysis.

**Results:**

A total of 7 studies were included. Spirometry was used in all included studies; however, it is unable to detect subclinical lung alterations, as observed through ventilation-perfusion (V/Q) MRI and fractional exhaled nitric oxide (FeNO). Acute exposure to EC results in a decrease of FEV₁, FVC, PEF, and MEF₇₅ spirometric parameters, as well as reducing FeNO levels, while concurrently increasing exhaled breath temperature (EBT). Besides, an increase in V/Q mismatch and heterogeneity in ventilation is observed, with a reduction in perfusion heterogeneity. Chronic EC exposure causes a reduction in FEV_1_, PEF, FEV_1_/FVC, and FEF_25–75%_, besides an increment of Carboxyhaemoglobin (HbCO) level. The assessment of the lung function post-EVALI in association with EC cessation revealed lung function improvement and increased diffusing capacity of the lung for carbon monoxide (DLCO).

**Conclusions:**

Spirometry remains the first-line tool for assessing the lung function of young EC users; however, it often misses early lung dysfunction. Emerging methods (FeNO, DLCO, EBT, MRI, HbCO) increasingly complement this limitation. Tailoring multimodal assessment to exposure context, alongside screening and monitoring programs, may assist in early disease detection and prevent long-term respiratory effects.

## Introduction

E-cigarettes (EC) were initially introduced into the market almost 30 years ago as one of the smoking-cessation initiatives to substitute the traditional cigarette [1]. Since then, EC use among youth and young adults has risen alarmingly [2]. This is because of perceived less harm, pleasing taste, and aggressive marketing campaigns of EC [3]. Nevertheless, EC users are exposed to aerosolized chemicals, including nicotine, propylene glycol, and other various irritants. Thus, evidence increasingly suggests that vaping is harmful and affects respiratory function [4]. Studies indicate that there is an increasing number of EC-related health effects cases, mainly E-Cigarette or Vaping Product Use-Associated Lung Injury (EVALI), especially among the young demographic [5,6]. Youth and young adults represent a critical developmental period where the drive for new experiences, such as substance use, predisposes them to conventional or electronic cigarette addiction, heightening the demographic’s vulnerability to its associated health risks [7–10].

Lung function comprises the pulmonary pump, the lung parenchyma, and pulmonary circulation [11]. Any of these components may be impaired due to certain pathologies, resulting in respiratory illnesses. Exposure to EC is known to impair respiratory structure and function among young EC users and EVALI patients, even without symptoms [12]. Current tools, such as spirometry, remain the primary method for assessing lung function. Yet, as research on EC expands, more emerging methods such as fractional exhaled nitric oxide (FeNO) are being utilised to detect more specific changes in lung function [13,14].

Youth and young adulthood are a critical period for lung growth. Thus, harmful exposures during a young age may have long-lasting effects on respiratory health, resulting in chronic lung disease [15]. A study indicates a significant association between EC usage as an independent risk factor with COPD and asthma development [16]. Hence, understanding the utilisation landscape of these lung function assessment tools and how they are applied is vital in examining the respiratory effects of EC among youth and young adult users. To the best of our knowledge, no prior systematic review has specifically examined lung function assessment tools used among youth and young adult e-cigarette users. Prior systematic studies on the health effects of EC usage are mostly concentrated on adult populations [17,18]. Additionally, the previous review on assessing the respiratory effects of EC was generalized to in vitro, animal, and human studies, without specifically addressing youth [19].

Addressing the gap, this review emphasizes the immediate need to understand and identify the tools used to assess lung function in this vulnerable population. Therefore, the objective of this systematic review was to examine the current and emerging methods used in assessing lung function in studies focused on EC exposure among youth and young adult populations. Besides, this study also aimed to identify the alterations in lung function following EC exposure among youth and young adults measured by those tools. Clarifying best practices, this review may guide healthcare practitioners in making informed clinical decisions for effective lung function assessment in young EC users. Additionally, this review may assist future researchers in designing the optimal method for evaluating lung function among young users of EC. Furthermore, this review corresponds with the fast development of EC technologies and emerging lung function assessment methods. Thus, highlighting its relevance in protecting the health of youth and young adults.

## Materials and methods

### Protocol and registration

This systematic review adheres to the Preferred Reporting Items for Systematic Reviews and Meta-Analyses (PRISMA) 2020 statement. The PRISMA 2020 checklist and the PRISMA 2020 Abstract checklist are included in **Supporting Information** S1 and S2 Appendix [20]. PROSPERO registration ID for this review is CRD420251138294.

### Eligibility criteria and research question formulation

The review question was developed using the PICOS (Patient/Population, Intervention, Comparator, Outcome, Study design) [21]. This concept was adapted, as this review will focus on identifying and describing the current tools and emerging methods of lung function assessment used for EC users, particularly among youth and young adults. In this review, the population refers to youth (15–24 years) and young adults (18–26 years) [22,23]. Intervention refers to lung function assessment tools; the comparator is not applicable in this review. The outcome is the preferred assessment tools and methods in assessing the lung function alterations among youth and young adults EC users in the study. Meanwhile, study types refer to any study design that reports on lung function assessments following EC exposure among young EC users. The PICOS concept guided the formulation of the review question: “Which current and emerging tools are frequently utilized to assess lung function in studies involving youth and young adult EC users?”. The second question is “What are the lung function alterations detected by the lung function assessment tools following EC exposure among youth and young adults?”.

### Data source and search strategy

The literature search was performed in April 2025 across six databases: Web of Science, PubMed, Scopus, Taylor & Francis, SAGE, and Science Direct. The keywords utilized for the search for related articles are presented in Table 1. The full electronic search strategy for all databases is provided in **Supporting Information** S1 File. From five databases, 550 related records were found. Using automated tools, 225 records were included according to publication type and year. For Science Direct, ten search strings with alternate keywords for Boolean strings were generated to search for related articles, resulting in 2662 related records found. Automated tools were used according to the publication type and year, excluding 1896 records. These articles from Science Direct were then screened for duplicates, and a total of 590 records were excluded from the list. All related records from six databases were combined, resulting in a total of 991 records. After a total of 623 duplicate records were discovered and eliminated, leaving 368 records for title screening. For screening, the records were exported from the databases into an Excel sheet.

**Table 1:** Keywords used in the screening process.

Database	Search string
Web of Science	TS=((“Electronic Nicotine Delivery System*” OR “Electronic Non Nicotine Delivery System*” OR “Electronic Cigarette*” OR “E-Cig*” OR “vape” OR “vaping”) AND (“lung function*” OR “pulmonary function test*” OR “pulmonary function*” OR “spirometry” OR “spiromet*”) AND (“youth” OR “adolescen*” OR “teenager*” OR “juvenile” OR “young adult*” OR “youngster*”))
PubMed	((“Electronic Nicotine Delivery System*” OR “Electronic Non Nicotine Delivery System*” OR “Electronic Cigarette*” OR “E-Cig*” OR “vape” OR “vaping”) AND (“lung function*” OR “pulmonary function test*” OR “pulmonary function*” OR “spirometry” OR “spiromet*”) AND (“youth” OR “adolescen*” OR “teenager*” OR “juvenile” OR “young adult*” OR “youngster*”))
Scopus	TITLE-ABS-KEY((“Electronic Nicotine Delivery System*” OR “Electronic Non Nicotine Delivery System*” OR “Electronic Cigarette*” OR “E-Cig*” OR “vape” OR “vaping”) AND (“lung function*” OR “pulmonary function test*” OR “pulmonary function*” OR “spirometry” OR “spiromet*”) AND (“youth” OR “adolescen*” OR “teenager*” OR “juvenile” OR “young adult*” OR “youngster*”))
Taylor&Francis	((“Electronic Nicotine Delivery System*” OR “Electronic Non Nicotine Delivery System*” OR “Electronic Cigarette*” OR “E-Cig*” OR “vape” OR “vaping”) AND (“lung function*” OR “pulmonary function test*” OR “pulmonary function*” OR “spirometry” OR “spiromet*”) AND (“youth” OR “adolescen*” OR “teenager*” OR “juvenile” OR “young adult*” OR “youngster*”))
SAGE	((“Electronic Nicotine Delivery System*” OR “Electronic Non Nicotine Delivery System*” OR “Electronic Cigarette*” OR “E-Cig*” OR “vape” OR “vaping”) AND (“lung function*” OR “pulmonary function test*” OR “pulmonary function*” OR “spirometry” OR “spiromet*”) AND (“youth” OR “adolescen*” OR “teenager*” OR “juvenile” OR “young adult*” OR “youngster*”))
Science Direct	1) ((“Electronic Cigarette” OR “E-Cig” OR “vape” OR “vaping”) AND (“lung function” OR “pulmonary function test” OR “pulmonary function “) AND (“adolescen” OR “young adult”)) 2) ((“Electronic Nicotine Delivery System” OR “Electronic Cigarette” OR “E-Cigarette”) AND (“lung function” OR “spirometry”) AND (“youth” OR “teenager” OR “young adult”)) 3) ((“Electronic Cigarette” OR “E-Cigarette” OR “vape”) AND (“lung function” OR “pulmonary function” OR “spirometry”) AND (“youth” OR “adolescent” OR “young adult”)) 4) ((“Electronic Cigarette” OR “E-Cigarette” OR “vape”) AND (“pulmonary function” OR “spirometry”) AND (“teenager” OR “juvenile” OR “youngster”)) 5) ((“Electronic Cigarette” OR “E-Cig” OR “vape”) AND (“lung function” OR “pulmonary function” OR “spirometry”) AND (“youth” OR “adolescent”)) 6) ((“Electronic Nicotine Delivery System” OR “Electronic Cigarette” OR “E-Cigarette”) AND (“lung function” OR “pulmonary function test” OR “pulmonary function “) AND (“adolescent” OR “young adult”)) 7) ((“Electronic Nicotine Delivery System” OR “Electronic Non-Nicotine Delivery System” OR “Electronic Cigarette”) AND (“lung function” OR “pulmonary function” OR “spirometry”) AND (“youth” OR “juvenile”)) 8) ((“Electronic Nicotine Delivery System” OR “Electronic Non-Nicotine Delivery System” OR “Electronic Cigarette”) AND (“lung function” OR “pulmonary function*” OR “spirometry”) AND (“adolescent” OR “young adult”)) 9) ((“Electronic Nicotine Delivery System” OR “Electronic Non-Nicotine Delivery System” OR “Electronic Cigarette” AND (“lung function” OR “spirometry”) AND (“youth” OR “adolescent” OR “young adult”)) 10) ((“Electronic Cigarette” OR “vape”) AND (“lung function” OR “pulmonary function” OR “spirometry”) AND (“teenager” OR “juvenile” OR “youngster”))

### Inclusion and exclusion criteria

The inclusion criteria were: (1) publication from 2016–2025; (2) original article; (3) publication in any language and country; (4) study assessing the lung function of electronic cigarette users among youth and young adults. We restrict the publication date to between 2016 and 2025 (original publications published during the last decade) to ensure our systematic review is based on current research, reflecting the significant evolution in EC usage and the devices themselves [24]. Non-original articles, including review articles and systematic reviews, case reports, conference proceedings, and commentary, were excluded.

### Study selection

Two authors (NIN and NA) independently screened the titles and abstracts according to the review questions. A total of 322 articles were removed during the screening. The remaining 46 articles were retrieved for assessment of eligibility. Disagreements were resolved by discussion with a third author (MHJ) to achieve consensus. Thirty-nine articles were excluded because of different study outcomes (n = 22), different populations, which were commonly on adults (n = 13), and animal studies (n = 4). Subsequently, the remaining 7 articles proceeded to quality appraisal.

### Risk of bias assessment

The quality of the 7 articles was assessed by 2 authors (NIN and MHJ). The articles were evaluated using the Joanna Briggs Institute (JBI) Critical Appraisal Tools. Given that studies included in this review adopt a range of quantitative study designs (e.g., cross-sectional and experimental studies) [25]. Thus, we chose these appraisal tools because we believe it is the best approach for this review, as they are flexible and robust for multiple study designs. The quality assessment was based on 8 items for the cross-sectional and 9 items for the quasi-experimental study [26,27]. Most included studies were assessed as having a low risk of bias. Majority adequately addressed key methodological domains such as reliable outcome measures and appropriate analysis. Only certain limitations were observed, mainly on controlling confounding factors, indicating overall strong confidence in the synthesized evidence. Completed checklists for all included studies are provided in **Supporting Information** S2 File.

### Data selection and data synthesis

**Fig 1:**
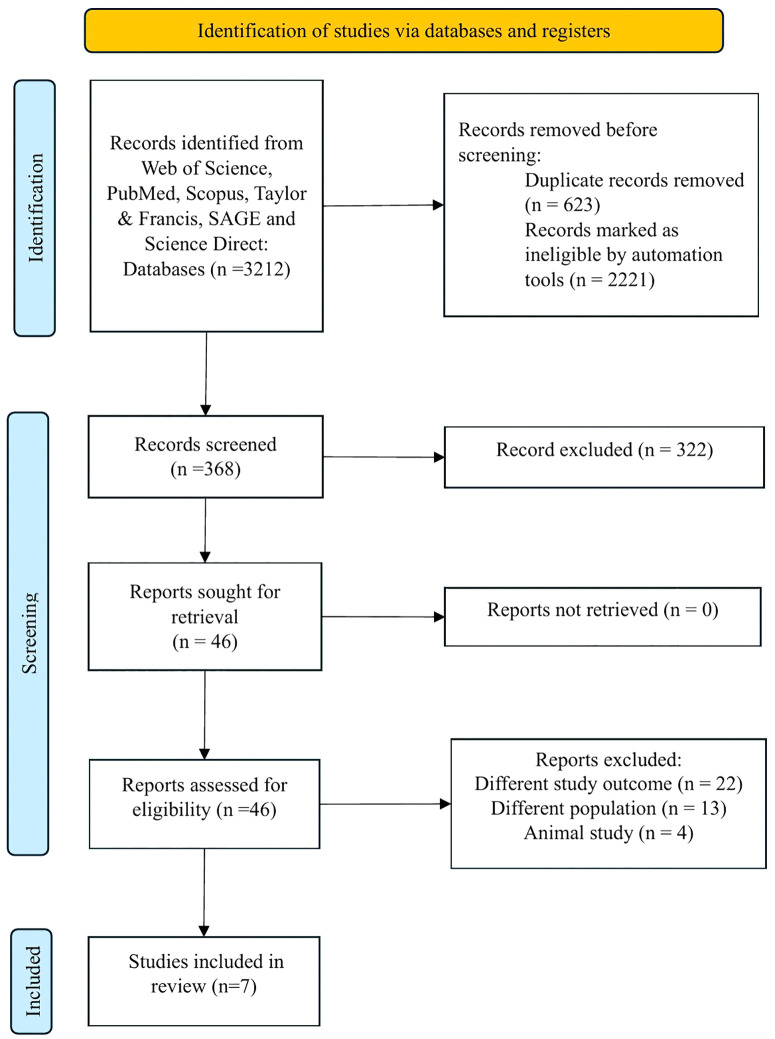
PRISMA flow diagram.

All authors independently gathered the data using a consistent data extraction form and arranged it in a standard Microsoft Excel file. Among the gathered facts and information were: (1) authors, (2) year of publication, (3) aim of study, (4) study location, (5) study design, (6) study population, (7) sample size, (8) lung function assessment tools, and (9) study outcome. **Fig 1** shows the PRISMA flow diagram. Narrative synthesis was chosen for this review based on the diverse and often heterogeneous nature of the included studies in terms of study design, outcome measures, and methodologies. This diversity means that pooling results quantitatively is not feasible.

## Results

### Background of the eligible studies

A total of 7 studies were included in this systematic review. A descriptive summary of the included studies is shown in **Table 2**. Studies included in this review were published between 2019 and 2023, with a notable increase in 2021. Study timeframe varies, as most of the studies (71.5%) were published within the last 5 years, while the remaining (28.5%) were published more than 5 years ago. Three studies were done in the United States of America (USA), two studies were conducted in the same state of Poland, while one study each was conducted in the United Kingdom (UK) and Sweden. Four papers involved a cross-sectional study approaching local subjects, such as university students. Three papers involved experimental studies, in which they recruited asymptomatic, healthy youth and young adults to undergo the intervention. Sample sizes range from 8 to 1000 samples.

**Table 2:** Characteristics of studies related to the assessment of lung function among youth and young adults who use e-cigarettes. (n = 7).

Author	Study location	Population	Study design	Sample size	Aim	Quality of study
Brożek et al 2019	Katowice, Poland	Healthy young adults: 4 groups of exclusive e-cigarette users (E-), dual users (T/E-), tobacco smokers (T-), and controls (C-)	Intervention study (pre-post-exposure)	120 total (30 per group: E-, T-, T/E-, C-)	To assess acute respiratory responses to using EC in exclusive EC users and dual users, and to compare these effects with responses to smoking tobacco cigarettes in tobacco smokers.	8/9
Darabseh et al. 2021	United Kingdom (Manchester Metropolitan University)	Healthy young adults (vapers, cigarette smokers, and non-users)	Cross-sectional study	44 total: vapers (n = 12), smokers (n = 14), controls (n = 18)	To assess and compare the effects of vaping and cigarette smoking on maximal respiratory pressures (MRP), respiratory function, and carboxyhaemoglobin levels.	6/8
Puliyakote et al. 2021	San Diego, California, USA (UC San Diego)	Asymptomatic young adult vapers vs. healthy non-vaping controls	Experimental pre-post imaging study	Vapers: 9, Controls: 7	To assess the impact of acute e-cigarette inhalation on ventilation-perfusion (V/Q) mismatch in asymptomatic vape users using proton MRI.	8/9
Lee et al. 2021	Texas Children’s Hospital and Baylor College of Medicine, Houston, USA	Adolescents diagnosed with EVALI	Cross-sectional study	8 patients	To evaluate pulmonary function recovery in adolescents with EVALI after discontinuing e-cigarette use.	7/8
Alhamdow et al. 2021	Sweden	Young adults from the Swedish cohort BAMSE study	Cross-sectional study	1000	To examine the association between low-level PAH exposure and lung function and respiratory inflammation in Swedish young adults.	6/8
Abdallah et al. 2022	Children’s Medical Center, Dallas, Texas, USA	Hospitalized adolescents diagnosed with EVALI	Cross-sectional study	41 (19 pre-pandemic, 22 during-pandemic)	To characterize clinical features and pulmonary function in adolescents hospitalized with EVALI before and during the COVID-19 pandemic.	5/8
Majek et al. 2023	Katowice, Poland	Healthy young adults (exclusive users of HTPs, e-cigarettes, traditional cigarettes, and controls)	Intervention study	160 total (40 per group: HTP, e-cigarette, cigarette, control)	To assess the acute effects of heated tobacco products and electronic cigarettes on respiratory and cardiovascular function in young adults.	8/9

The aim of the studies varies, with three investigating acute changes in lung function parameters before and after EC use using laboratory-based designs, in comparison with other groups such as non-smokers, conventional cigarette smokers, and dual users. Meanwhile, one study focuses on imaging modalities to assess the impact of acute EC inhalation on ventilation-perfusion (V/Q) mismatch in asymptomatic users using proton MRI. Another study with a cross-sectional design concentrated on polycyclic aromatic hydrocarbons and their relationship with lung function among young EC users. Another study compares respiratory function and maximal respiratory pressures between young EC users, smokers, and non-smokers. Furthermore, two studies observed the progression of lung function of discharged EVALI patients through subsequent outpatient follow-up

### Lung function assessment tools used

**Table 2** shows tools used to assess lung function. Spirometry was used in all studies. The most parameters reported by Spirometry were FEV₁, FVC, and FEV₁/FVC ratio. It is an established lung function assessment tool and has become the first line and reference, even among the youth and young adults, for researchers. The second most used instruments were fractional exhaled nitric oxide (FeNO) and Exhaled Carbon Monoxide (CO which were employed by three studies. Exhaled breath temperature and the diffusing capacity of the lung for carbon monoxide (DLCO) were employed by two studies. Meanwhile, Maximal Respiratory Pressures (MRP) & Sniff Nasal Inspiratory Pressure (SNIP), Carboxyhaemoglobin (HbCO), and MRI Ventilation-Perfusion Imaging were used once among all 7 included studies. All the researchers in the included studies are seen to employ multiple lung function assessment methods along with spirometry as a first-line tool.

### Lung function assessment across exposure settings

The EC exposure settings range from acute laboratory interventions to chronic usage and post-treatment follow-up. Four studies assessed the lung functions of youth and young adults with chronic exposure to EC, while 3 studies assessed lung functions in acute exposure to EC. All 3 acute EC exposure studies were experimental, and the alterations of lung function were evaluated and compared between baseline and post-exposure. Two studies examining chronic EC exposure recruited participants from general populations and educational institutions, with eligibility requiring a minimum of one year of EC use. Another two studies focused on young patients with chronic usage of EC and were diagnosed with EVALI. These studies assessed improvements in lung function during post-discharge follow-up in relation to the discontinuation of EC use.

### Lung function alterations across exposure settings

Acute exposure studies demonstrated measurable alterations of lung functions on a few assessment tools. Alike changes were detected by 2 studies following acute exposure to EC, with changes detected by spirometry, FeNO, and exhaled breath temperature. There was a reduction of spirometric parameters (one study shows a reduction of FEV_1_ and FVC, another study shows reduced PEF and MEF_75_). Besides, both studies revealed a reduction of FeNO level and a significant increase in exhaled breath temperature following acute EC exposure. On the other hand, both studies revealed that acute exposure to EC did not significantly alter Exhaled CO. A study employed advanced imaging, MRI Ventilation-Perfusion Imaging, to assess lung function alteration post-acute EC exposure. In this study, a notable increase in ventilation-perfusion (V/Q) mismatch and heterogeneity in ventilation is observed, alongside a reduction in perfusion heterogeneity. However, despite these changes, spirometry remains normal upon acute EC exposure.

A study on chronic EC exposure assessment among young EC users revealed a reduction of FEV_1_, PEF, FEV_1_/FVC, and FEF_25–75%_ on spirometry and an increased level of HbCO. However, no significant changes were detected on MRP and SNIP. A study among chronic young vapers with PAH exposure revealed a reduction in FEV_1_ and FVC, but no significant alteration in FeNO level. Another two studies focus on the assessment of the lung function alteration post-EVALI admission among youth and young adult EC users. Both studies revealed identical findings of improvement of spirometric parameters and increased DLCO, associated with EC cessation. Alterations of lung function detected by the tools in the included studies were summarised in **Table 3**.

**Table 3:** Assessment and exposure setting, assessment tools, and findings of included studies.

Author	Assessment tools	Exposure setting		Findings
		Acute	Chronic	
Brożek et al 2019	• Spirometry • FeNO • Exhaled breath temperature • Exhaled CO	Assessment at Baseline, 1-minute post-use, 30-minute post-use.	–	• Reduced PEF, MEF_75_ • Decreased FeNO • Increased exhaled air temperature • No significant in Exhaled CO
Puliyakote et al, 2021	• MRI Ventilation-Perfusion Imaging • Spirometry	Immediate imaging after vaping.	–	• Acute vaping increased V/Q mismatch and heterogeneity in ventilation. • Perfusion heterogeneity decreased after vapping • Spirometry remained normal
Majek et al. 2023	• Spirometry • FeNO • Exhaled breath temperature • Exhaled CO	Assess lung function at baseline, immediately, and after 30 minutes of exposure.	–	• Decreses in FEV_1_ and FVC. • Reduced FeNO post-exposure for e-cigarette. • Increase in exhaled breath temperature. • No significant in Exhaled CO.
Darabseh et al 2021	• Spirometry • Carboxyhaemoglobin (HbCO) • Maximal Respiratory Pressures (MRP) & Sniff Nasal Inspiratory Pressure (SNIP)	–	Participant with a history of daily E-cigarette usage for 1 year or more.	• Reduced FEV_1_, PEF, FEV_1_/FVC, FEF_25–75%_ • Higher HbCO • MRP & SNIP does not significantly differ
Alhamdow et al. 2021	• Spirometry • FeNO	–	Measure the association between urinary PAH and lung function among young chronic EC users.	• Dose-response relationship between urinary PAH metabolites and reduced FEV_1_ and FVC. • No association with FeNO
Lee et al. 2021	• Spirometry • Diffusing capacity of carbon monoxide (DLCO)	–	Assessment of lung function post-EVALI during clinic follow-up with association with EC discontinuation.	• Spirometry significantly improved from moderate impairment to normal • Improvement of DLCO (increased) • Recovery linked to discontinuation of vaping
Abdallah et al. 2022	• Spirometry • DLCO	–	Assessing improvement of lung function among EVALI patients before discharge and during follow-up.	• Improved FEV_1_ and FVC post-treatment and upon follow-up • DLCO improved (increased)

## Discussion

### Research landscape on lung function assessment among youth and young adults E-Cigarette users

A marked rise in studies of lung function assessment among young EC users was observed in 2021. This uptick aligns with growing global concerns about EC-related lung injuries, particularly after the EVALI outbreak in the United States of America [28]. The outbreak has garnered significant attention due to its extensive media coverage and increased global awareness [29]. Thus, this urge has been a significant epidemiological turning point, attracting interest in assessing the respiratory effects of EC, particularly among young users. There is deep concern among healthcare providers regarding this issue. Responsive shift by them to re-evaluate the perceived safety of EC has increased research funding and prioritization, mainly among the younger demographic [30].

The evolving legal and regulatory response post-EVALI outbreak has prompted the research activity. By early 2020, the United States Food and Drug Administration (FDA) banned certain EC products due to their commitment to limit respiratory risks, especially among young users [31]. Besides, the government of India implemented a nationwide ban on the production, import, and sale of EC [32]. Mexico also prohibits the import of EC and heated tobacco products into the country during the post-EVALI outbreak [33]. This surge is linked with the strategies in strengthening legal actions to protect the young generation from harmful EC exposure globally. Redirection of scientific and public health studies, focusing on examining EC’s impact on health, is needed to support regulatory justifications and risk assessments [34].

A noticeable clustering of studies was observed in specific countries. This may be attributed to their strong laws regulating EC use, particularly among the young demographic. Poland and Sweden’s compliance with the EU Tobacco Products Directive (TPD) 2017 ranges from national product notification systems, age restrictions, advertising prohibitions, and excise duties [35,36]. Tobacco and Related Products Regulations 2016 (TRPR), in the United Kingdom, establishes comprehensive rules for EC manufacture, labelling, nicotine limits, packaging, and product notification [37]. Meanwhile, in the US, regulations are robust, spanning federal, state, and local levels. The FDA Authority & Premarket Approval (PMTA) gained authority to regulate tobacco products, including EC. In addition, the Minimum Legal Sales Age (Tobacco 21), which was enacted in 2019, raised the minimum age for purchasing vaping products to 21, with no exceptions [38,39]. Strong and well-enforced legislation in these countries creates the right conditions for researchers to assess and evaluate the policy. Thus, generating evidence to inform regulatory improvements and protecting public health, especially that of the young generation.

### Lung function alteration in acute exposure to E-cigarettes

Acute exposure studies have demonstrated that even short-term exposure to EC leads to measurable changes in lung function among youth and young adults. Fractional exhaled nitric oxide (FeNO) is prominent in detecting significant alteration of lung function following acute EC exposure. It is a non-invasive airway inflammatory marker, usually linked with eosinophilic activity [13]. Conditions such as asthma, allergic responses, or irritant exposure from pollutants induce eosinophilic airway inflammation. Therefore, inducible nitric oxide synthase (iNOS) activity increases, producing more Nitric oxide (NO). This NO diffuses into the airway lumen and can be detected in exhaled breath as FeNO. This review found a significant reduction in FeNO level following acute EC exposure among youth and young adults. This finding is consistent with previous literatures, which examine the lung function among adults and asthmatic patients upon acute EC exposure [40–42]. The reduction of FeNO may be explained by the oxidative stress consuming nitric oxide (NO) or the transient suppression of iNOS activity post-acute EC exposure [43,44].

This review also reveals that increased exhaled breath temperature (EBT) was seen as a response to acute EC exposure. This tool has been proposed as a proxy marker for airway inflammation, particularly in asthma and chronic obstructive pulmonary disease (COPD) [45,46]. It is primarily determined by the temperature of the airways and the metabolic processes occurring within the lungs. The increment of EBT may be linked to the thermal properties of the aerosol produced due to the heating of e-liquid. Higher temperatures then lead to increased combustion, generating free radicals, thus contributing to airway inflammation and changes in EBT [41,47]. The observed increment in EBT post-acute EC exposure is consistent with a prior study reporting a similar derangement in lung function among second-hand EC smokers [48].

Another tool that significantly detects lung function alteration after acute EC exposure is MRI Ventilation-Perfusion Imaging. This is consistent with a previous review that indicates MRI Ventilation-Perfusion Imaging as a valuable tool for examining lung function associated with acute EC exposure [49]. This tool enables non-invasive assessment of regional lung function by capturing airflow and blood flow distribution patterns among EC users. Comparison between ventilation and perfusion maps that reveal any mismatch mandates acute functional impairment. This method is significant in the early detection of subclinical lung abnormalities, where current approaches, such as spirometry, remain insensitive. This method provides a sensitive regional measure of lung dysfunction, facilitating early diagnosis and further thought on the pathophysiology associated with EC exposure [50,51].

### Lung function alteration in chronic exposure to E-Cigarettes

Chronic exposure to EC in youth and young adults manifests more severely, mainly as EVALI. In the context of chronic EC exposure, spirometry remains the primary method for detecting alterations in lung function. Components of spirometry, such as FVC, FEV_1_, PEF, FEV_1_/FVC, and FEF_25–75%_, were found to be significantly altered following chronic exposure to EC. It is indicated by a reduction in their measurements. These findings are in alignment with previous literature studied among adults and coal miners in chronic EC exposure [44,52]. Besides, this review further emphasized that longitudinal spirometry is able to reveal critical information on the reversibility of lung function. A few studies examine the lung function among EVALI patients during post-discharge follow-up, revealing positive outcomes on spirometry evaluation. A case report earlier stated the same finding for an EVALI patient with an 8-month history of daily EC usage, with complete resolution of symptoms and pulmonary function [53]. Such effects are most notable among youth and young adults, with complete discontinuation of EC. However, this finding contrasts with a previous study, which reported that smoking cessation activity did not reverse the respiratory function [54].

In addition, this review found that DLCO has a role in detecting lung function alteration following chronic EC exposure among youth and young adults. This tool assesses gas exchange efficiency at the alveolar-capillary membrane. An isolated reduction in DLCO is regarded as an emerging early diagnostic marker for both pulmonary parenchymal and vascular pathologies [55]. This observed finding is aligned with a study that reported a lower DLCO among healthy, asymptomatic individuals, with at least 2 years history of EC usage [56]. This might be due to loss of gas‑exchange surfaces, indicating damage to functioning alveolar units after chronic EC exposure [57].

### Emerging methods supporting and complementing current tools in lung function assessment

This review revealed the role of FeNO and EBT in supporting spirometry in detecting lung function alterations among youth and young adults post-EC exposure. In these studies, the reduction in spirometry parameters is accompanied by a decrease in FeNO and an increase in EBT measurement. The main spirometry parameters that show a significant reduction are FEV_1_, FVC, PEF, and MEF_75_ [58,59]. The observed finding is consistent with a prior study among asthmatic patients reporting a significant association between measurement of FeNO and spirometry [60]. However, this observation diverges from a study where no significant correlation was found between EBT and spirometric indices among paediatric patients [61].

Puliyakote et al. employed proton MRI to detect ventilation-perfusion (V/Q) mismatches for acute EC exposure in their interventional study. Although used in fewer studies due to resource intensity, MRI ventilation-perfusion mismatch assessments offer an advanced, non-invasive means of assessing regional lung function. In an acute setting, spirometry appeared not to be able to detect lung function alteration. While MRI revealed increased V/Q mismatch and perfusion heterogeneity, spirometry showed normal values. This might be due to its insensitivity in detecting disease during the initial stages. Besides, spirometry is also not sensitive in determining regional variability and gradual functional lung changes [50,51]. This finding is consistent with a previous study that suggested spirometry alone is not sufficient to diagnose interstitial lung disease in patients with early diffuse cutaneous systemic sclerosis [62]. However, emerging methods such as MRI suggest that EC usage induces subtle microvascular or ventilation irregularities [63]. As a supplement to current modalities of pulmonary function assessment, functional lung imaging has the potential to identify respiratory disease phenotypes with distinct natural histories. Thus, this points to the limitations of relying solely on spirometry in detecting lung function alterations among youth and young adults with EC exposure.

Carboxyhaemoglobin (COHb) is a complex formed in the blood when carbon monoxide (CO) binds to haemoglobin with an affinity approximately 200–250 times greater than oxygen [64]. It serves as a biomarker of chronic CO accumulation, reflecting sustained exposure from sources. The chronic accumulation of CO may affect airway function and obliterate alveoli, thus altering lung function [65]. The circulating COHb mostly originates from exogenous exposure, such as conventional and EC smoking, as well as vehicle exhaust. Once CO is inhaled, it reaches the alveoli, crosses the alveolar-capillary membrane, and binds to the iron-containing site of hemoglobin [64]. A study among chronic EC users revealed that HbCO level is significantly related to the duration of smoking exposure and nicotine intake [66]. A study on HbCO measurement among young adult users with a 1–2-year history of EC usage shows an increase in COHb compared to non-EC users [54]. Thus, chronic EC exposure is likely to be significant for CO accumulation, at the same time, complementing the spirometric finding.

### Limitations of tools in detecting lung function alteration

Several tools employed in the included studies showed no detectable alteration in lung function following EC exposure. Exhaled CO is produced endogenously in healthy, non-smokers, elevated in several inflammatory lung conditions [67]. However, it may originate from exogenous sources such as smoking, air pollution, and allergic reactions [68–70]. This review revealed no alteration in exhaled CO measurement among young EC users. This is supported by a study that found no significant correlation between exhaled CO levels and lung function reduction among petrol station attendants [71]. This might be due to low CO emission by ECs compared to conventional cigarettes [72]. Maximal Respiratory Pressures (MIP/MEP) & Sniff Nasal Inspiratory Pressure (SNIP) are non-invasive tools that may assist in respiratory muscle strength assessment [73]. This can be explained by a study that reported no correlation between respiratory muscle strength and lung spirometry testing among vapers and multiple sclerosis patients [74,75]. This finding suggests that reduced airflow due to EC exposure is due to airway obstruction rather than reduced respiratory muscle strength.

### Potential reversibility of lung function impairments

Post-discharge clinic follow-up of young EVALI patients further indicated that lung function could improve following treatment and discontinuation of EC use. This stresses the potential reversibility of impaired lung functions due to harmful EC exposure. The outcomes supported a study that revealed repeated pulmonary function tests on all their EVALI patients showed resolved abnormalities within 1 year of hospital discharge [76]. This points out that lung function gains were observed in a relatively short period after the impairment, emphasizing the physiological capacity for rapid recovery in younger populations. However, these findings are in contrast with a previous study that indicates a persistent residual airway reactivity or diffusion abnormalities among adolescents EVALI patients during follow-up [77]. Therefore, this finding suggests that some EC-induced lung impairments may not be permanent and potentially reversible with early treatment and EC discontinuation.

### Recommendation

Translating existing evidence into practice, these recommendations aim to optimise screening and diagnostic approaches for lung function alteration associated with EC use among youth and young adults. In acute EC exposure, initial evaluation may include spirometry and FeNO as first-line tools in detecting early functional and inflammatory alterations. Moreover, EBT may be considered as an adjunct tool; meanwhile, advanced modalities such as MRI ventilation–perfusion imaging should be reserved for selected cases. This method may be utilized in cases where other assessments are inconclusive, and the clinical necessity justifies the higher cost-to-benefit ratio [78]. Measurement during presentation, particularly in cases with a history of acute exposure (within minutes to 1 hour), is crucial. Meanwhile, repeated testing beyond the acute phase is generally not required unless symptoms persist or recur. This is due to most studies indicating that acute changes are transient and reversible [58,59,79].

Individuals with chronic EC exposure, routine monitoring of lung function using spirometry, FeNO, HbCO, and DLCO, where available, together with regular symptom assessment, is considered appropriate. Baseline measurement should be recorded with repeated measurements during follow-up, typically every 6–12 months, depending on symptom progression, trend of EC use, and the physician’s clinical judgment [80,81].

Moreover, for suspected or confirmed EVALI cases, baseline measurement at presentation using spirometry and DLCO is applicable if the patient is clinically stable. Alternatively, assessments may be deferred to the early recovery phase, allowing evaluation of post-exposure functional changes [81,82]. This should be followed by serial reassessments during the recovery period to evaluate the resolution or persistence of functional impairment. Short-term follow-up is recommended within 1–8 weeks post-discharge, with additional reassessment at 4–6 months, particularly in individuals with persistent abnormalities [83,84]. Repeated testing is important to document functional recovery, residual deficits, or progression of impairment.

### Public health implication

This review may provide significant public health implications, particularly among the youth and young adults. Timely screening among EC users and proper diagnostic strategies for EVALI using appropriate lung function assessment tools based on their exposure setting are crucial for early intervention. This is linked to the potential benefit of timely cessation strategies and early treatment, particularly in younger populations whose lung tissues retain greater resilience compared to older adults. Besides, there is still limited longitudinal data on the long-term effects of EC. Thus, this review carries an important public health agenda in reinforcing the EC cessation programme into adolescent and youth primary care plans. Furthermore, wider governmental and public health initiatives, such as policies and regulations, are needed to reduce EC use in this age group.

### Strengths and limitations

This review is limited by the relatively small number of included studies in view of the limited research available, particularly among youth and young adults. Consequently, the results of this review may have limited generalizability. Moreover, variations in study designs and population characteristics may introduce potential heterogeneity that could not be fully accounted for. Confounding factors across the included studies, such as former conventional cigarette users, environmental exposures, and pre-existing respiratory illness, were not controlled. This thereby limits the strength of causal interpretations.

The strength of this review is the focus of the study on youth and young adults, a high-risk group, where the evidence is still emerging, making the synthesis timely and relevant. Besides, this review systematically compares both current and emerging lung function assessment methods, with multiple exposure settings covered. By including acute and chronic exposure assessments, this review provides a relatively complete picture of lung function alterations across different exposure contexts with varied assessment tools used. Besides, this review is relevant to public health and clinical practice. By linking findings to implications for early screening, EC cessation interventions, and monitoring in youth and young adults, this review provides practical insights beyond academic value. Finally, the use of JBI tools to evaluate the study quality ensures methodological transparency and strengthens the credibility of this review.

### Future research directions

Future research should expand the study on the assessment tools in comparison with different populations, such as older adults. Besides, studies on different tools used among different geographical and socioeconomic backgrounds, as well as ethnic groups, may improve external validity and help understand disparities in outcomes. Besides, future studies are warranted to thoroughly design the study to properly control the confounders.

## Conclusion

Spirometry remains the first-line lung function assessment tool used in assessing lung function among youth and young adults EC users. However, it has limitations, especially in detecting early alterations of lung function. Consequently, it has increasingly been complemented by emerging assessment methods. Tools such as FeNO, MRI, exhaled breath temperature, HbCO, and DLCO detect the alterations based on inflammatory reactions and imaging studies. Lung function alterations have been significantly observed in acute and chronic exposure to EC. Lung function assessment of post-discharge EVALI patients has shown remarkable improvement, linked to EC discontinuation. Thus, utilising proper tools based on users’ exposure is important in detecting significant changes in lung function, providing the young generations with the benefits of advanced technological assessment tools. After all, systematic youth and young adult programs on vaping cessation, screening, and monitoring are prompted to prevent the harmful effects of EC, ensuring better health for future generations.

## Supporting information

S1 Appendix The PRISMA 2020 checklist. (PDF)

S2 Appendix The PRISMA 2020 Abstract checklist. (PDF)

S1 File Search strategy. (DOCX)

S2 File Completed Joanna Briggs Institute (JBI) Critical Appraisal Checklists for each included study. (ZIP)
