# Supplementary material for: Systematic review of lung function assessment among youth and young adults e-cigarette users: Current tools and emerging methods
Source: PLoS One. 2026 Feb 6;21(2):e0342500. doi: 10.1371/journal.pone.0342500 (PMC12880674; doi:10.1371/journal.pone.0342500)
Supplement: S1 File — (DOCX) [file pone.0342500.s003.docx]

**Search strategy**

| Objective | 1. To examine the current and emerging methods used in assessing lung function in studies focused on E-Cigarette exposure among youth and young adult populations. 2. To identify the alterations in lung function following E-Cigarette exposure among youth and young adults, as measured by those tools. |
| --- | --- |
| Literature type | Published articles |
| Study settings | Global |
| Inclusion criteria | (1) Publication from 2016-2025  (2) Original article  (3) Publication in any language  (4) study assessing the lung function of E-cigarette users among youth and young adults. |
| Exclusion criteria | Non-original articles, including review articles and systematic reviews, case reports, conference proceedings, and commentary. |
| Databases | Web of Science, PubMed, Scopus, Taylor & Francis, SAGE, and Science Direct |
| Languages | Any languages |
| Search period | Searches were performed in April 2025 |

**Database search terms**

| **Database** | **Keywords** | **Filters** |
| --- | --- | --- |
| Web of Science | TS=(("Electronic Nicotine Delivery System*" OR "Electronic Non Nicotine Delivery System*" OR "Electronic Cigarette*" OR "E-Cig*" OR "vape" OR "vaping") AND ("lung function*" OR "pulmonary function test*" OR "pulmonary function*" OR "spirometry" OR "spiromet*") AND ("youth" OR "adolescen*" OR "teenager*" OR "juvenile" OR "young adult*" OR "youngster*")) | (1) Year: 2016-2025  (2) Document type: article |
| PubMed | (("Electronic Nicotine Delivery System*" OR "Electronic Non Nicotine Delivery System*" OR "Electronic Cigarette*" OR "E-Cig*" OR "vape" OR "vaping") AND ("lung function*" OR "pulmonary function test*" OR "pulmonary function*" OR "spirometry" OR "spiromet*") AND ("youth" OR "adolescen*" OR "teenager*" OR "juvenile" OR "young adult*" OR "youngster*")) | (1) Year: 2016-2025  (2) Document type: article |
| Scopus | TITLE-ABS-KEY(("Electronic Nicotine Delivery System*" OR "Electronic Non Nicotine Delivery System*" OR "Electronic Cigarette*" OR "E-Cig*" OR "vape" OR "vaping") AND ("lung function*" OR "pulmonary function test*" OR "pulmonary function*" OR "spirometry" OR "spiromet*") AND ("youth" OR "adolescen*" OR "teenager*" OR "juvenile" OR "young adult*" OR "youngster*")) | (1) Year: 2016-2025  (2) Document type: article |
| Taylor&Francis | (("Electronic Nicotine Delivery System*" OR "Electronic Non Nicotine Delivery System*" OR "Electronic Cigarette*" OR "E-Cig*" OR "vape" OR "vaping") AND ("lung function*" OR "pulmonary function test*" OR "pulmonary function*" OR "spirometry" OR "spiromet*") AND ("youth" OR "adolescen*" OR "teenager*" OR "juvenile" OR "young adult*" OR "youngster*")) | (1) Year: 2016-2025  (2) Document type: article |
| SAGE | (("Electronic Nicotine Delivery System*" OR "Electronic Non Nicotine Delivery System*" OR "Electronic Cigarette*" OR "E-Cig*" OR "vape" OR "vaping") AND ("lung function*" OR "pulmonary function test*" OR "pulmonary function*" OR "spirometry" OR "spiromet*") AND ("youth" OR "adolescen*" OR "teenager*" OR "juvenile" OR "young adult*" OR "youngster*")) | (1) Year: 2016-2025  (2) Document type: article |
| Science Direct | 1) (("Electronic Cigarette" OR "E-Cig” OR “vape" OR "vaping") AND ("lung function" OR "pulmonary function test" OR "pulmonary function ") AND ("adolescen" OR "young adult"))  2) (("Electronic Nicotine Delivery System" OR “Electronic Cigarette" OR "E-Cigarette") AND ("lung function" OR "spirometry") AND ("youth" OR "teenager” OR "young adult”))  3) ((“Electronic Cigarette" OR "E-Cigarette" OR “vape") AND ("lung function" OR "pulmonary function" OR "spirometry") AND ("youth" OR "adolescent” OR "young adult"))  4) (("Electronic Cigarette" OR "E-Cigarette” OR “vape") AND ("pulmonary function" OR "spirometry") AND ("teenager" OR "juvenile" OR "youngster"))  5) (("Electronic Cigarette" OR "E-Cig” OR "vape") AND ("lung function" OR "pulmonary function" OR "spirometry") AND ("youth" OR "adolescent"))  6) (("Electronic Nicotine Delivery System" OR “Electronic Cigarette" OR "E-Cigarette") AND ("lung function" OR "pulmonary function test" OR "pulmonary function ") AND ("adolescent" OR "young adult"))  7) (("Electronic Nicotine Delivery System" OR "Electronic Non-Nicotine Delivery System" OR "Electronic Cigarette") AND ("lung function" OR "pulmonary function" OR "spirometry") AND ("youth" OR "juvenile"))  8) (("Electronic Nicotine Delivery System" OR "Electronic Non-Nicotine Delivery System" OR "Electronic Cigarette") AND ("lung function" OR "pulmonary function*" OR "spirometry") AND ("adolescent" OR "young adult"))  9) (("Electronic Nicotine Delivery System" OR "Electronic Non-Nicotine Delivery System" OR "Electronic Cigarette" AND ("lung function" OR "spirometry") AND ("youth" OR "adolescent" OR "young adult"))  10) (("Electronic Cigarette" OR "vape") AND ("lung function" OR "pulmonary function" OR "spirometry") AND ("teenager" OR "juvenile" OR "youngster")) | (1) Year: 2016-2025  (2) Document type: article |
